# The Challenges of Measuring and Valuing Quality of Life in Preschool Children: A Retrospective Review of NICE Appraisals

**DOI:** 10.3390/children8090765

**Published:** 2021-08-31

**Authors:** Alan Lamb, Alice Murray, Rosie Lovett

**Affiliations:** Science Policy and Research, National Institute for Health and Care Excellence (NICE), Level 1A, City Tower, Piccadilly, Manchester M1 4BT, UK; alice.murray@nice.org.uk (A.M.); rosemary.lovett@nhs.net (R.L.)

**Keywords:** health technology assessment, utilities, health-related quality of life, preschool children, patient reported outcome measures

## Abstract

Health technology assessment agencies evaluate interventions across the lifespan. However, there is no consensus about best-practice methods to measure health-related quality of life (HRQoL) in preschool children (<5 years) and data are often scarce. We reviewed methods used to capture the HRQoL of preschool children in past National Institute for Health and Care Excellence (NICE) appraisals to establish whether there is a need for better methods in this area and if so, to identify priority research areas. We identified past NICE appraisals that included preschool children, examining the methods used to generate utility values and whether committees believed these captured HRQoL adequately. Of the 12 appraisals, most used generic HRQoL measures designed for adults. Measures were usually completed by adult patients or clinical experts. Committees frequently commented on limitations in the HRQoL data. While acknowledging that data collection may be challenging, committees would value evidence based on HRQoL data from parents or guardians collected as part of a clinical trial. We identified several research priorities including the psychometric properties of existing measures; the feasibility and validity of valuation studies; and mapping. Progress in these areas will help ensure that the aspects of HRQoL which matter to children and their families are captured in NICE evaluations.

## 1. Introduction

The health-related quality of life (HRQoL) of children and young people is often not measured in trials. Consequently, age-appropriate utility data are rarely available to inform economic models. One reason for paediatric utility data not being collected is a lack of methods guidance from either health technology assessment (HTA) agencies or the literature. 

In the context of measuring health-related quality of life in children, NICE’s ‘Guide to the methods of technology appraisal 2013’ states that when necessary, consideration should be given to alternative measures to EQ-5D (which is the preferred measure of health-related quality of life in adults). These alternatives should be “standardised and validated preference-based measures of health-related quality of life that have been designed specifically for use in children.” [1]. A previous systematic review of UK paediatric cost–utility analyses found that the methods used for utility analysis were highly variable, and differed from the NICE reference case more often than other elements of the analysis [2]. Further reviews of paediatric cost-utility analyses which include studies from outside of the UK have found variations in methodological practice similar to those reported in the UK review, suggesting that other HTA agencies face similar challenges to NICE [3,4]. NICE is currently working on providing clearer guidance, including clarifying the desired properties for generic preference-accompanied instruments (that is, a health state descriptive system that is accompanied by a set of health state utility values) that measure children and young people’s quality of life. NICE’s updated guidance is informed by a systematic review of the psychometric properties of several measures undertaken by NICE’s Decision Support Unit [5]. 

However, clearer guidance on use of preference-accompanied instruments is uninformative for studies of preschool children (defined in this report as children under five years), as most measures are designed for children over the age of five. Therefore, NICE is not currently able to provide explicit methods recommendations for this age group, and to the best of our knowledge we are not aware of any HTA organisations which provide such recommendations. Furthermore, there are additional challenges when measuring and valuing HRQoL for preschool children, including the need for proxy measurement, whether measurement scales should focus on observable behaviour and accounting for rapid developmental change.

We reviewed past NICE appraisals that included preschool children to identify the methods and sources used to generate health state utility values and examine whether these were seen by the committee and evidence review group (ERG) to adequately capture the HRQoL of young children. We then used the findings of this review to identify priority research areas which may support the generation of age-appropriate evidence for preschool children.

## 2. Methods

This work builds upon a previous review, published by Hill et al., of health state utility values in NICE evaluations for people aged under 18, which included guidance published before November 2019 [6]. The Hill et al. review did not examine subgroups by age. The current review identified the subgroup of appraisals where children aged under five years were either included in the scope of the appraisal or were not explicitly outside of the marketing authorisation of the technology (24 of 40 of the appraisals identified in Hill et al.). To bring the search up to date, NICE technology appraisal (TA) and highly specialised technology (HST) guidance published from November 2019 to August 2020 was manually searched to identify additional evaluations including the target age group [7]. Guidance prior to April 2004 was excluded as this predated the existence of the NICE methods guide for technology appraisals. Consequently, pre-2004 appraisals tend to use less common methodological approaches; including these early appraisals could lead to outdated approaches being overrepresented in a relatively small data set.

In our analysis, we assessed which measure was used (e.g., EQ-5D or HUI3), who completed it (e.g., children, parents or clinicians) and how it was valued (that is, how the information about health-related quality of life was converted into utility scores on the scale where one is perfect health and zero is equivalent to dead). This information was drawn from the appraisal documents including final and draft guidance, company submission, ERG/assessment group report, technical reports and responses to consultation. In addition, descriptive data were extracted to identify themes related to: The company and ERG’s rationale for their preferred method, committee comments on the strengths and limitations of the chosen method and any additional comments relevant to the preschool population. 

Having extracted the data, we observed that some appraisals had a broad age range (e.g., two to 18 years) but the bulk of the population was over the age of five, meaning that the evidence base and committee discussions focussed on these older children. Accordingly, we selected a final analysis set that included only those appraisals where the utility values used for preschool children were likely to be important in the context of the appraisal. The decision to include an appraisal in the final analysis set also took into account the natural history of the disease, whether preschool children were a distinct subgroup in the economic model, whether specific recommendations were made for preschool children, and other relevant comments in the appraisal documentation. Deciding which appraisals to include was a subjective process, and therefore two researchers (AL and AM) independently assessed whether appraisals should be included, and any discrepancies were resolved by consensus.

We also identified how many appraisals included infants (defined as children under one) and examined whether improved HRQoL evidence specifically for this age group would be likely to be important in the context of the appraisal.

## 3. Results

### 3.1. Included Appraisals and Summary of Results

**Figure 1:**
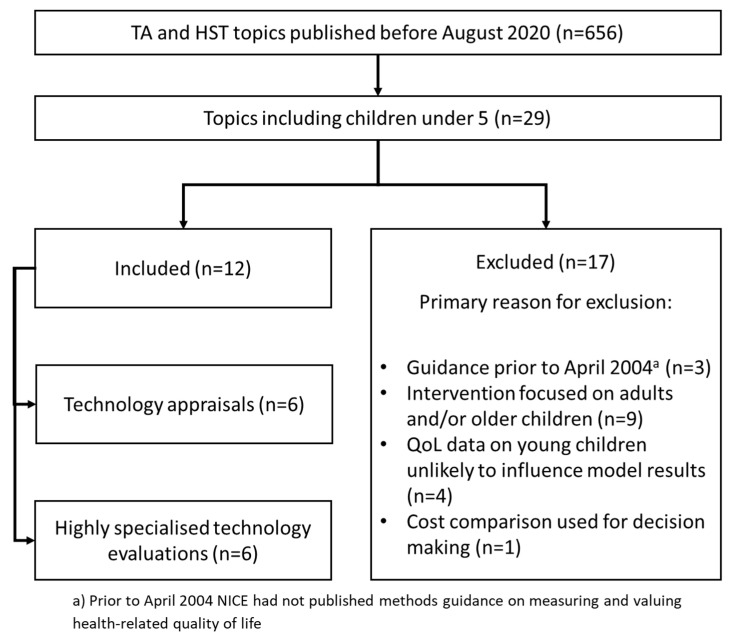
Number of topics in analysis set and primary reasons for exclusion.

A total of 29 appraisals whose population included children under five were identified. A total of 12 topics, comprising six TAs and six HSTs, were included in the final analysis set (see Figure 1).

In NICE’s standard single technology appraisal process, the company is responsible for identifying the evidence to inform the health state utility values used in the economic model. This evidence may come from data collected in the company’s trials or from the literature (preferably from a systematic review). The ERG may identify additional evidence during their critique of the company’s submission and suggest alternative utility values. Consequently, the committee may discuss several possible health state utility values. Our analysis focussed on the methods used to calculate the most influential health state utility values in the committee’s preferred model (that is, the model used for decision-making). We identified the measure used to derive utility values, the type of study where the measure was collected and who completed it, and who completed the valuation tasks used to convert response data into utility values. The results are summarised in Table 1.

### 3.2. Measures of HRQoL Used to Derive Health State Utility Values in Models

**Figure 2:**
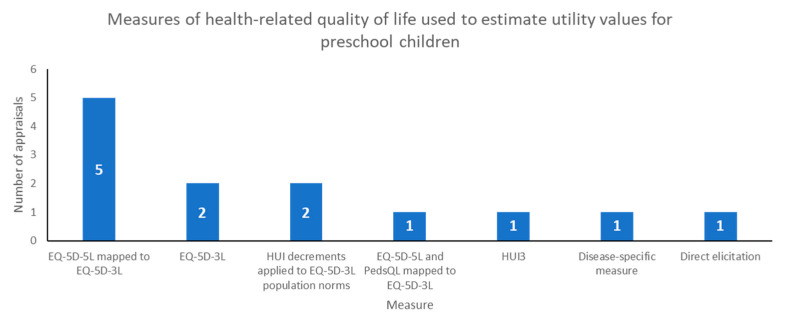
Measures used in NICE appraisals to estimate utility values for preschool children.

Nearly all the included appraisals used a preference-accompanied generic measure of quality of life, in line with the NICE reference case for adult utility values (see Figure 2). In HST11, the committee considered two sets of utility values without deciding which was preferred, so the number of methods sums to 13.

The most common measure used was EQ-5D-3L: either mapped from EQ-5D-5L, n = 6; used directly, n = 2; or general population values with utility decrements calculated from the HUI, n = 2. In one appraisal where the company performed mapping from EQ-5D-5L to EQ-5D-3L (HST11), the committee also considered utility values from a disease-specific preference-accompanied measure identified by the ERG. One appraisal (TA630) included pooled data from multiple measures (EQ-5D-5L and PedsQL) mapped to EQ-5D-3L. HUI3 was used directly in TA566 because the condition involved hearing loss, where the HUI may be more sensitive than EQ-5D [20]. One appraisal (TA588) used direct elicitation of utility values from experts. It should be noted that this was not the initial approach proposed by either the company or ERG, but was an attempt to resolve differences in opinion around the face validity of utility values proposed in certain health states in the model.

We also examined supplementary evidence that did not inform the economic model. Evidence from generic measures of quality of life designed for either preschool children or older children was identified by either the company or ERG in eight of the 12 appraisals. However, many of these measures are not preference-accompanied (or do not have UK value sets). Only one generic measure (PedsQL) was identified in more than one appraisal, indicating a lack of strong preference for the use of any single measure designed for children. Evidence from disease-specific measures was only identified in one TA but was common in HST evaluations, with four of the six evaluations identifying evidence from such measures.

### 3.3. Who Is Completing the Measure and in What Kind of Study?

Across these diverse appraisals, one unifying factor is that the utility values were rarely based on evidence directly from preschool children or their parents or guardians. These data were only presented in three of the 12 included appraisals (25%) and even in these cases did not necessarily cover the full population being evaluated, as can be seen in Table 1 for TA566. Instead, preschool utility values usually relied on data from studies including adults or older children with the condition (in the case of TAs) or the interpretation of health states by clinical experts (in the case of HSTs). The main reason companies gave for the use of adult data is that limited (or no) HRQoL data were available for preschool children. On some occasions, companies advised that the model was not suitable for use in a paediatric population or that inclusion of separate utilities for young children would overcomplicate the model.

**Figure 3:**
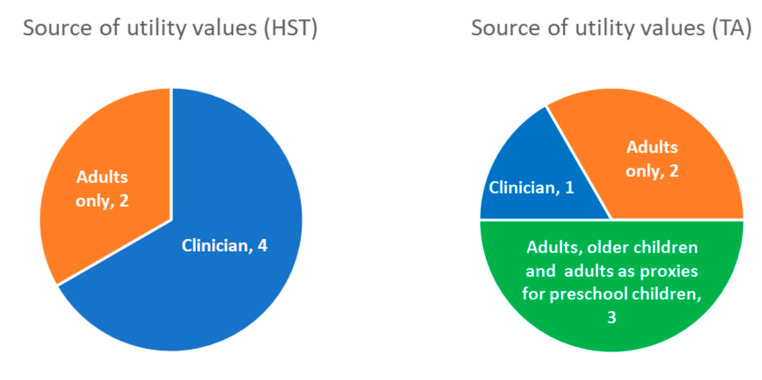
Sources of utility values used for preschool children in NICE appraisals.

The data sources used by HSTs and TAs are distinct and therefore it is informative to consider these separately (see Figure 3). The majority of HST evaluations (4/6) used ‘vignette’ studies, where respondents were given descriptions of the health states in the model and were asked to complete a questionnaire (usually the EQ-5D-5L or 3L) based on these descriptions. In all cases, the questionnaires were completed by clinicians experienced in treating the condition. One appraisal (HST2) used data from a burden of illness survey completed by children, adults and parents or guardians of young children, but only the values from adults were used in the economic model. The remaining appraisal (HST7) derived health state utilities by applying utility decrements to EQ-5D general population norms. These utility decrements were based on literature data for adverse effects in other conditions, which were considered suitable proxies for the condition being appraised.

Only three TAs used utility values derived from studies which included preschool children. However, there were limitations to the approach used in each of these. This paper does not report full details of who completed the measure because these data either were not reported in the company submission or were marked as confidential by the company. In one appraisal (TA630), data from children aged under 2 years old were excluded because there was no suitable mapping algorithm available to convert responses from the PedsQL infant scale to EQ-5D-3L values. One further TA (TA566) had two distinct clinical populations, one of which used data from studies including preschool children and the other data from adults only. Finally, TA538 used utility decrements derived from the HUI, which requires use of a non-UK value set. Of the three remaining TAs, two used data from studies which included adults only and one used directly elicited expert clinical opinion.

In the included TAs, most health state utility values were derived from observational studies identified in the literature (4/6). Only one appraisal (TA630) used utility values derived from the pivotal clinical study of the intervention (in this case a single-arm trial). In TA588, the health state utility values used in the company’s final model were based on direct elicitation from experts, as discussed above.

### 3.4. How the Measures Were Valued

Two-thirds of appraisals in the analysis (8/12) used the UK EQ-5D-3L value set to generate utility values from questionnaire response data. This means that the valuation task was performed by adult members of the UK general public, although in one case (HST11) the committee also considered data from a disease-specific measure valued by members of the general public in several countries including the UK. In one appraisal (TA151) the health states were derived using a sample of UK diabetes patients who completed time trade-off valuation tasks. Utility values derived from the general public of other countries were used in two appraisals; in one case (TA566) because the HUI3, which has no UK value set, was used as the utility measure; and in the other (TA300) because the EQ-5D data from other countries were based on a larger sample size and were more recent than the available UK data. In the second case, the committee noted that it would have preferred UK data, but felt that its availability would not have significantly altered the conclusions of the cost-effectiveness analysis. The remaining appraisal (TA588) used utility scores directly elicited from experts.

### 3.5. Themes from Committee Conclusions

NICE appraisal committees are aware that “identifying robust utility values in babies and young children is exceptionally challenging” (TA588). We identified several themes from the committee conclusions in the final appraisal documentation of the 12 included appraisals. The main themes identified (present in three or more appraisals) were: a lack of or limitations in the quality of life data (9/12, 75%), high uncertainty in the utility values (5/12, 42%), health-related benefits which were not captured by the utility values (5/12, 42%), that the utility values used were appropriate or likely to be the best available (4/12, 33%), that the approach of using adult values was not appropriate/validated (3/12, 25%) and that the committee would have preferred to have seen utility values based on clinical trial data (3/12, 25%). In the four appraisals where adult data were exclusively used to generate utility values, the committee only indicated that these data were suitable on a single occasion, otherwise stating that such an approach was either unsuitable or not validated. All three instances of the committee stating a preference for trial data are from HSTs where the company had also presented vignette studies, indicating committee concerns around the limitations of vignette studies even in the context of very rare conditions and a young patient population. 

When committees indicated that health-related benefits were not captured in the utility values they took this into account in their decision making. For example, in TA566 the committee noted that bilateral cochlear implants may increase opportunities for interaction and communication for deaf children. This includes opportunities to participate in play activities, and benefits from education, which were unlikely to be captured in the cost-effectiveness model because it used utility values derived from adults.

### 3.6. Appraisals including Infants

**Figure 4:**
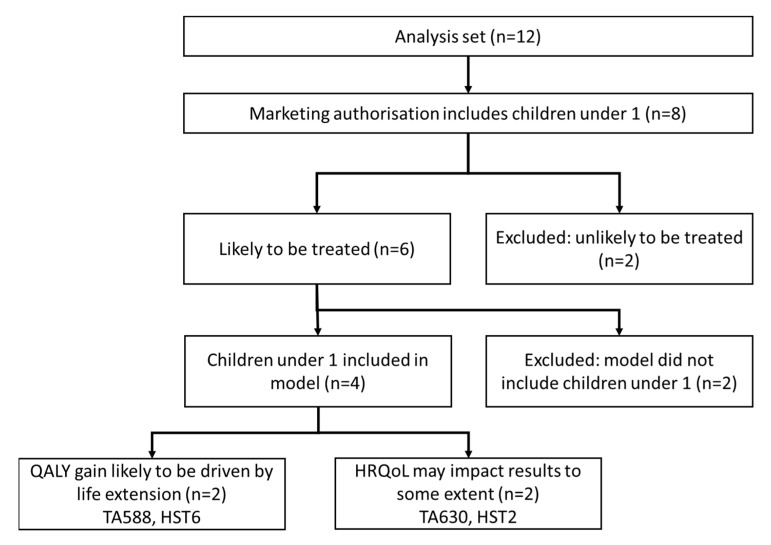
NICE appraisals including children under 1 as part of the population.

Several appraisals included technologies with marketing authorisations which include infants (defined here as children under one). For the technologies where infants were likely to receive treatment 4/6 included them in the economic model (see Figure 4). In two of these appraisals gains in QALYs are likely to be driven by life extension rather than quality of life (meaning the QALYs gained in early life are likely to be a small proportion of the total QALYs gained for life-saving interventions, see case study of HST6 below). In the remaining two appraisals it is plausible that HRQoL evidence generated specifically for babies and infants may influence the results, but the extent of any impact is unclear and may be small compared with other uncertainties in the evidence base.

## 4. Case Study: Differences across the Preschool Age Range

One of the challenges of measuring HRQoL for preschool children is that different considerations may apply at different ages due to a young child’s rapid development and/or a condition where the disease course is age dependent. An illustrative example is HST6, asfotase alfa for treating paediatric-onset hypophosphatasia. Hypophosphatasia is a genetic condition which disrupts the mineralisation process, where calcium and phosphorus are deposited in bones and teeth. Hypophosphatasia leads to a range of symptoms including rickets, weakening of the bones, bone deformity and fractures. There are several clinical forms depending on the age of onset of the condition, and people who present with perinatal- or infantile-onset hypophosphatasia (onset in the first six months of life) have a high mortality rate. 

Asfotase alfa is a targeted enzyme replacement therapy designed to restore the regulation of metabolic processes in the bones and teeth, and to reduce complications associated with hypophosphatasia. The committee noted asfotase alfa is a life-saving intervention for infants, whereas for children diagnosed above one year of age, the main benefit of treatment is improving quality of life rather than length of life. This influenced the economic modelling: including infants in the model led to a larger gain in the total number of quality-adjusted life years for children treated with asfotase alfa. This gain was largely driven by an increase in life years gained, that is the number of QALYs gained because of improved quality of live in the first year of life is very small compared with QALYs gained from additional years of survival; therefore, the model’s estimated utility values for the first year of life cannot substantially affect the results. 

The committee concluded that “the benefits of asfotase alfa in people with perinatal- and infantile-onset hypophosphatasia were of a different nature to those in the older groups, and that they were also both the largest and the least uncertain in this population group” and took this into account when making its decision. In this example, additional data on quality of life for infants would not substantially influence the cost-effectiveness estimates because the results are driven by life years gained rather than quality of life. In contrast, if better data on quality of life were available for children aged older than one year this may have reduced uncertainty in the modelling of the older preschool age groups. For example, quality of life data in this model were obtained by having clinicians complete vignette studies. NICE’s proposed updated methods expresses a preference for data from clinical trials—or, if vignettes must be used, for them to be scored by members of the public, parents/guardians or carers. 

Asfotase alfa was recommended for use with a managed access agreement. In addition to providing the treatment with a discount and agreeing data collection to reduce some of the uncertainties in the evidence base, the managed access agreement also set out the patient population which would be eligible to receive alfotase alfa. Infants diagnosed with hypophosphatasia are automatically eligible for treatment. For older children (where there are greater uncertainties in the evidence base), there are several stopping and starting criteria, one of which is quality of life as measured by the PedsQL scale. This highlights that having a reliable way to measure the quality of life of young children is not just a niche concern of economic modelling, but also has important clinical uses and may be employed in managed access agreements. It is therefore important that the psychometric properties and feasibility of measures designed for preschool children are evaluated with all these potential uses in mind.

## 5. Discussion

HTA agencies such as NICE are responsible for evaluating treatments for preschool children, and these are among the most challenging, emotive and controversial appraisals that they do. Our review found a high proportion of NICE HSTs and a small proportion of TAs include preschool children. An informal analysis of upcoming TA and HST topics shows that the number including preschool children is either steady or slightly increasing over the next few years. Thus, this age group should not be ignored in research into methods of measuring and valuing health-related quality of life.

Gathering good-quality data on health-related quality of life is challenging for all children [21,22], but doing so for preschool children brings its own unique difficulties [23]. The developmental changes from birth to five years are incredibly rapid [24,25]. Furthermore, because young children are unable to complete questionnaires themselves, these must be completed by proxy. FDA and ISPOR taskforce recommendations state that the focus of questionnaires should be on “observable” outcomes to minimise the influence of subjective judgements made by the proxy [26,27]. However, these recommendations were written from the perspective of the data required for licensing. From an HTA perspective, there is concern that focussing on “observable” outcomes may mean that the measure focuses on physical functioning and does not capture all elements of HRQoL which are important to the child. Further research into how these non-observable elements could best be captured by proxy reports may be valuable, but the research would need to involve decision-makers to consider whether the broader information on quality of life was sufficiently valid and reliable. Another challenge is that it is not possible to fully separate the influence of the carer-child dyad upon each other [28,29,30,31]. A seriously unwell child is likely to negatively affect the parent’s health-related quality of life, and vice versa. 

Given these difficulties, and the lack of consensus on best practice in the academic literature [21,22,23], it is perhaps not surprising that NICE does not have explicit guidance on measuring and valuing health-related quality of life in preschool children (whilst we have not conducted an exhaustive review, we do not believe other HTA agencies do, either). The present review found that almost all NICE appraisals in this age group used a preference-accompanied generic measure of quality of life, typically EQ-5D. None of the measures used in these appraisals has been demonstrated to be suitable for use in preschool children, so their validity, reliability and sensitivity in this age group are unknown. Regarding who completed the measure, for HSTs, the most common approach was to gather data from clinical experts who read vignettes of health states; for TAs the data often came entirely or primarily from adults with the condition. This difference probably reflects the paucity of data to inform typical HST evaluations, because the conditions are very rare, because adult patients may not be similar to child patients, and in some cases because there is little adult data to extrapolate from as patients may not survive into adulthood.

Our review reveals that committees were frequently concerned about limitations in the quality of life data and the resulting uncertainty in the utility values. Although the sample is small, two conclusions emerge. First, committees would prefer to see data from children or their parents and guardians, rather than just from adults with the condition or clinical experts. Second, HST committees would prefer to see data from clinical trials rather than vignettes alone. This latter finding indicates that, while there may be limitations of utility values derived from clinical trials for rare disease which include preschool children, committees would find it useful to see such data alongside vignette studies to inform their conclusions on the validity of and uncertainty around the utility values used in the economic model. Overall, and by necessity, NICE committees take a pragmatic approach and weigh up all the available evidence in a process of deliberative decision-making. 

Our review only identified a small number of appraisals where babies and infants were included in the economic model and in half of these it was judged that mortality was the main driver of QALY gains. We speculate that even when quality of life is a key driver of the model, the time period from birth to first birthday is so short and the challenge of gathering HRQoL data so substantial that committees may well be content to use data from slightly older children to inform utility values for infants. Thus, on balance, research into methods designed for infants could currently be less of a priority for NICE than research into methods for children over the age of one.

Proposed changes to the NICE methods guide [32,33,34] do not focus on preschool children. Nonetheless, the proposed update provides more direction on relevant methods than has been available previously, including

(a) Support for measuring the health-related quality of life of children and young people using a generic measure—provided it has good psychometric performance in the relevant age range(s).
(b) Support for clearer reporting of who is completing the measures and how it is valued. Proxy reporting should be by carers rather than professionals.
(c) A hierarchy of preferred methods for measuring health-related quality of life, including advice on when disease-specific measures may be considered. The draft methods guide states “If there is evidence that generic measures are unsuitable for the condition or intervention, refer to the hierarchy of preferred sources for health-related quality of life.” [34] The hierarchy also includes general information on the design and conduct of vignette studies which is applicable to young children [35,36].

Suggested research priorities include:

(a) Research on the psychometric properties of generic measures when applied to this age group (for example; known-group validity, content validity, face validity, reliability and responsiveness). Candidate measures include those initially designed for older children and those specifically designed for the under-five’s, for example several have been identified in systematic reviews [37,38,39] or have been recently published [40].
(b) Research into the feasibility and validity of valuation studies of those measures.
(c) Research into the feasibility and validity of mapping from measures used in preschool children onto measures intended for older children or adults (this may be useful if the population includes a mix of preschool and older children).

The key strengths of this study are that it provides a comprehensive overview of NICE technology evaluations of preschool children over the past 16 years. Quantitative data were supplemented by an exploration of the rationale behind the choice of utility values and the committee’s critique of these data. Key limitations are a small sample size and a focus on NICE technology evaluations. Thus, we cannot know whether our conclusions are generalisable to other countries or to clinical guidelines.

## 6. Conclusions

This study examined the methods and data sources used to generate utility values for NICE evaluations of preschool children. We found a reliance on data from adults or from clinical experts. Even when data on the quality of life of preschool children were collected, the instruments used were not validated in this population. These limitations reflect a shortage of age-appropriate measures and a lack of consensus about best-practice methods for this age group. However, this paper is not a counsel of despair. NICE committees are well accustomed to being presented with uncertain evidence, and their deliberative approach ensures that limitations in evidence and methods are not a barrier to patients accessing innovative treatments. Nonetheless, there is room for improvement, and we highlight key areas for further research. Progress in these areas will help ensure that the aspects of quality of life which matter to young children and their families are captured in NICE evaluations.

## Figures and Tables

**Table 1:** Methods used to measure and value quality of life in NICE appraisals that included preschool children.

Appraisal (Year)	Condition	Main Measure(s) Used to Derive Utility Values	Where Measure Was Collected	Who Completed Measure	Who Completed Valuation Tasks
TA151 (2008) [8]	Diabetes	EQ-5D-3L	Observational study	Adults with the condition	UK patients with diabetes
TA300 (2013) [9]	Hepatitis C	EQ-5D-3L	Observational study	Adults with the condition	Non-UK general public (Swedish and Canadian value sets)
TA538 (2018) [10]	Neuroblastoma	HUI used to calculate % decrement applied to EQ-5D-3L general population values	Observational study	Adults and older children with the condition. Adult proxy completion for preschool	% decrements: Non-UK general public (Canadian HUI value set) General population norms: UK general public
TA566 (2019) [11]	Cochlear implants (update of TA166, 2009)	HUI3	Observational studies	Unilateral implant populations: adults and older children with the condition. Adult proxy completion for preschool children Bilateral implant population: adults with the condition	Non-UK general public (HUI 3 Canadian value set)
TA588 (2019) [12]	Spinal muscular atrophy	Direct elicitation of utility values from clinical experts	N/A	N/A	N/A
TA630 (2020) [13]	NTRK mutation positive cancer	EQ-5D-5L mapped to 3L and PedsQL mapped to EQ-5D-Y (applying adult value set)	Clinical trial	Adults and older children with the condition. Adult proxy completion for preschool children	UK general public (UK EQ-5D-3L adult value set)
HST2 (2015) [14]	Mucopolysaccharidosis type IVa	EQ-5D-5L mapped to 3L	Burden of illness survey	Adults with the condition Data collected for older children with the condition were not used in the model	UK general public (UK EQ-5D-3L adult value set)
HST6 (2017) [15]	Hypophosphatasia	EQ-5D-5L mapped to 3L	Vignette study	Clinicians	UK general public (UK EQ-5D-3L adult value set)
HST7 (2018) [16]	Adenosine deaminase deficiency/severe combined immunodeficiency	Utility decrement from HUI applied to EQ-5D-3L general population values	Observational study	Adult proxy completion for older children with a similar condition.	Utility decrement: Non-UK general public (HUI 3 Canadian value set) General population norms: UK general public
HST8 (2018) [17]	X-linked hypophosphataemia	EQ-5D-5L mapped to 3L	Vignette study	Clinicians	UK general public (UK EQ-5D-3L adult value set)
HST11 ^a^ (2019) [18]	Retinal dystrophy (RPE65 gene mutations)	Company: EQ-5D-5L mapped to 3L ERG: Disease-specific preference-accompanied measure (Visual Function Questionnaire-25)	Company: Vignette study ERG: Time trade-off study	Company: Clinicians ERG: Adults (general public)	Company: UK general public (UK EQ-5D-3L adult value set) ERG: general public (UK, US, Australia and Canada)
HST12 (2019) [19]	Neuronal ceroid lipofuscinosis type 2	EQ-5D-5L mapped to 3L	Vignette study	Clinicians	UK general public (UK EQ-5D-3L adult value set)

^a^ The committee stated that the utility values proposed by the committee and ERG were both considered and that ‘true’ utility values were likely somewhere between the two approaches.

## Data Availability

The appraisals analysed for this article are publicly available on the NICE website at https://www.nice.org.uk/guidance/published (accessed on 26 August 2021).
